# Correction of Two Papers: “Functional Cross-Talk between the α_1_- and β_1_-Adrenergic Receptors Modulates the Rapidly Activating Delayed Rectifier Potassium Current in Guinea Pig Ventricular Myocytes” and “Population Structure of the Greenhouse Whitefly, *Trialeurodes vaporariorum* (Westwood), an Invasive Species from the Americas, 60 Years after Invading China” in *Int. J. Mol. Sci.* Volume 15, Issue 8, 2014

**DOI:** 10.3390/ijms150915766

**Published:** 2014-09-05

**Authors:** IJMS Editorial Office

**Affiliations:** Klybeckstrasse 64, Basel 4057, Switzerland; Tel.: +41-61-683-7734; Fax: +41-61-302-8918; E-Mail: ijms@mdpi.com

Two papers [1,2] were recently published with incorrect page range, DOI numbers and publishing date in the PDF full text versions. For [1], the correct page range is 14220–14233, the correct DOI number is 10.3390/ijms150814220, and the correct publishing date is 15 August 2014. For [2], the correct page range is 13514–13528, the correct DOI number is 10.3390/ijms150813514, and the correct publishing date is 4 August 2014. We apologize for any inconvenience to the authors or readers.
